# Estimating Area of Occupancy From Incomplete Occurrence Records: Reproducible Workflow Illustrated on a Comprehensive Dataset of Georgian Snakes

**DOI:** 10.1002/ece3.74292

**Published:** 2026-09-03

**Authors:** Giorgi Iankoshvili, David Tarkhnishvili

**Affiliations:** ^1^ Leibniz Center for the Caucasus Biodiversity, Institute of Ecology Ilia State University Tbilisi Georgia

**Keywords:** area of occupancy, breakpoint analysis, data‐deficient species, IUCN Red List, sampling bias, scale‐dependency, sensitivity analysis

## Abstract

Area of occupancy (AOO) is a widely used metric for assessing species' vulnerability. To standardize AOO estimation, the IUCN recommends using a fixed 2 × 2 km grid. However, for species represented by sparse and irregular records, this resolution can substantially underestimate AOO. Although spatial modeling offers a potential solution, model‐based estimates may differ among taxa and studies depending on data structure, predictor choice, and modeling strategy. In this paper, we present a reproducible workflow that uses breakpoint analysis of record‐accumulation curves to identify informative species‐specific grid sizes and to estimate AOO from incomplete occurrence records. We applied this framework to 13 snake species found in Georgia, with diverse ecological characteristics within a topographically complex, irregularly sampled region. Repeated subsampling of the six best‐represented species showed that accumulation‐curve predictions were more accurate than raw occupied‐cell counts in 96%–98% of comparisons. Nine of the 13 species showed significant breakpoint‐derived scales between approximately 4 and 13 km rather than at the standard 2 km resolution. This workflow offers a practical way to explore scale dependence, sampling incompleteness, and sensitivity of AOO estimates derived from historical and citizen‐science occurrence datasets. The workflow complements the standardized 2 × 2 km AOO used in formal IUCN Red List assessments.

## Graphical abstract

The workflow is presented that helps to optimize grid‐cell size by breakpoint search and to estimate the area of occupancy (AOO) more precisely, if the occurrence data are incomplete. The model was validated on 13 colubrid snakes found in Georgia. The scripts in R and full statistical workflow are provided in the paper and the Supporting Information.

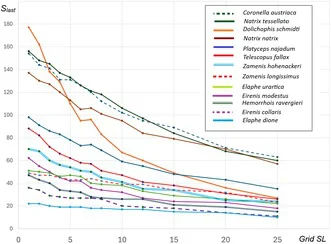

**Abbreviations**
AOO: area of occupancy
*C*
: sample completeness, *S*_last_/*S*_max_
EOO: extent of occurrence
GPS: global positioning system
IUCN: the International Union for Conservation of Nature
*n*
: the number of records of a species
SL: grid‐cell size length
*S*_last_
: the number of occupied grid cells
*S*_max_
: saturation level/asymptotic number of occupied grid cells
*S*(*n*): the number of occupied grid cells given *n* records
UTM: universal transverse Mercator coordinate system
*ψ*
: breakpoint‐derived grid cell size for inferring AOO

## 1 Introduction

The area of occupancy (AOO) is one of the most important metrics used to infer species vulnerability at global and regional scales (IUCN 2024). The standard approach for estimating AOO is to calculate a cumulative area based on the number of grid cells with side length (SL) 2 km where a species is present. However, for species that are small and easy to overlook, such as amphibians, reptiles, small mammals, and most invertebrates, the total number of geographically distinct records of a species may strongly underrepresent its actual range and obscure uncertainty in current range knowledge. Imperfect detection underestimates true occupancy (MacKenzie et al. 2002). One possible way to address this problem is to model suitable habitats of a species using predictors potentially related to its occurrence. Anderson (2023) suggested combining empirical datasets with spatial modeling. This approach is reasonable, but it may introduce errors due to subjective selection of predictors, non‐representative datasets, or the absence of a species from some potentially suitable areas. In this paper, we analyze the potential of an alternative approach, based on the idea to estimate the asymptotic number of occupied grid cells from incomplete occurrence records. Along with using a standard 2 km grid, we propose evaluating a dataset‐specific breakpoint‐derived grid size at which occupied‐cell counts become less sensitive to spatial resolution, thereby providing an additional basis for estimating AOO from incomplete occurrence data. The proposed workflow is reproducible and helps to estimate model‐derived AOO while evaluating sampling incompleteness and sensitivity to the historical sequence of occurrence records.

This study examines whether analyzing saturation curves may complement the IUCN‐recommended workflow and improve knowledge of the area of occupancy of small animals, particularly when distribution data for the target species are incomplete and do not cover their entire range. For the analysis, we used a dataset on the records of 13 Colubrid snakes from Georgia (Bannikov et al. 1977; Tarkhnishvili et al. 2002; Tuniyev et al. 2020). The focal taxa represent contrasting geographic and ecological patterns. *Natrix natrix* , *Natrix tessellata* , *Coronella austriaca* , and *Zamenis longissimus* are broadly distributed in western Eurasia; *Elaphe dione* has a broad Central and East Asian distribution, whereas the remaining species are primarily associated with the eastern Mediterranean and West Asian region (IUCN 2026). Within Georgia, *Natrix* spp. are widespread but strongly associated with aquatic habitats; *Z. longissimus* and *C. austriaca* are mainly mesophilic; *Eirenis collaris* is restricted to semiarid landscapes; and several other species are associated predominantly with dry and/or rocky habitats (Nikolsky 1913; Muskhelishvili 1970; Bannikov et al. 1977). Some of these snakes, although not necessarily rare, are easily overlooked even during intensive sampling, so their documented ranges remain strongly dependent on sampling effort. Differences in body size, habitat use, and detectability further contribute to heterogeneous sampling completeness among species.

For over 100 years of herpetological studies in Georgia (the information summarized in Nikolsky 1913; Muskhelishvili 1970; Bannikov et al. 1977; Tarkhnishvili et al. 2002), Colubrid snakes were recorded in tens of sites; however, they were scarcely distributed or, conversely, concentrated in the areas commonly visited by professional herpetologists. Thanks to the rapid development of specialized web resources, social media, digital photography and mobile applications that enable rapid recording of precise geographic coordinates, the number of recordings has increased exponentially in recent years. Iankoshvili and Tarkhnishvili (2021) analyzed the available information from specific publications since the early 20th century onwards, including their personal records and hundreds of sites identified by amateur naturalists. They found that the number of records substantially increased for different snake species since the mid‐2010s, and commonly doubled in a very short period, related to the century‐long studies of professional zoologists. The study by Iankoshvili and Tarkhnishvili (2021) led to a substantial increase in the empirically confirmed extent of occurrence (EOO) and areas of occupancy (AOO) of multiple snake species, including Colubrids and Vipers. However, the effect of accumulating records on estimated AOO depends not only on their number but also on their spatial arrangement relative to previously known occurrences. New records close to established localities may fall within already occupied cells, whereas geographically distant records are more likely to add new occupied cells; the strength of this effect itself depends on grid size (Hui 2009; Azaele et al. 2012; Barwell et al. 2014). In our dataset, the recent increase in records was accompanied by an expansion of geographically documented occurrence sites (Iankoshvili and Tarkhnishvili 2021), so the accumulation of occupied cells reflects both the number and the spatial distribution of new records.

Here, we infer the AOO of snake species by analyzing the dynamics of the cumulative number of occupied grid cells of different sizes based on real data from 13 Colubrid species in Georgia. We offer a simple and reproducible workflow to determine a breakpoint‐derived grid‐cell size at which occupied‐cell counts become less sensitive to spatial resolution and to estimate AOO from the resulting accumulation curves. To evaluate the performance of this approach directly, we additionally used repeated random subsampling to test whether saturation curves fitted to incomplete occurrence datasets could predict the number of occupied cells subsequently documented in the complete empirical datasets. The breakpoint‐derived estimates are intended as complementary measures for evaluating sampling incompleteness and scale dependence in addition to formal IUCN AOO assessments using the standardized 2 × 2 km reference grain.

## 2 Materials and Methods

### 2.1 Workflow Summary

1. Organizing the information as a spreadsheet including geographic coordinates of each new finding and cumulative number of grid cells of different sizes.
2. Inferring breakpoint‐derived grid‐cell size by breakpoint analysis.
3. Calculating parameters of the saturation curves/fitting saturation curves and estimating asymptotic number of occupied grid cells (*S*_max_).
4. Calculating extent of occurrence (EOO) and area of occupancy (AOO).
5. Hold‐out validation and record‐order sensitivity.

### 2.2 Sources of Information on the Snake Localities and Organizing the Information

The published scientific papers that contain the information on the snake records in Georgia were summarized in Iankoshvili and Tarkhnishvili (2021). Starting in 2017, we monitored social media networks (Facebook, Instagram, iNaturalist, and Twitter). In addition, amateur naturalists and students (see the Acknowledgements section) regularly shared relevant information from their Facebook sites with the first author. For each photographed record posted on social media, we contacted the contributor to obtain locality details. If GPS coordinates were unavailable, we requested information sufficient to locate the site within ±1 km accuracy.

All known information on the occurrence localities of the following 13 Colubrid snake species was compiled and tabulated: ( *N. natrix* [L., 1758]), dice snake ( *N. tessellata* [Laurenti, 1768]), smooth snake ( *C. austriaca* [Laurenti, 1768]), European cat snake ( *Telescopus fallax* [Fleischmann, 1831]), ring‐headed dwarf snake ( *Eirenis modestus* [Martin, 1838]), collared dwarf snake ( *E. collaris* [Ménétries, 1832]), red‐bellied racer ( *Dolichophis schmidti* [Nikolsky, 1909]), Dahl's whip snake ( *Platyceps najadum* [Eichwald, 1831]), spotted whip snake ( *Hemorrhois ravergieri* [Ménétries, 1832]), Urartian rat snake (*Elaphe urartica* [Jablonski et al., 2019]), steppe rat snake ( *E. dione* [Pallas, 1773]), Aesculapian snake ( *Z. longissimus* [Laurenti, 1768]), and Transcaucasian rat snake ( *Zamenis hohenackeri* [Strauch, 1873]). The complete occurrence dataset used in the analyses, including species identity, locality, geographic coordinates, date used for chronological ordering, and broad source category (bibliographic or field/citizen‐science), is provided as Supporting Information S1.

The complete occurrence dataset used in the analyses included full‐precision geographic coordinates. For conservation reasons, the version provided as Supporting Information S1 contains spatially generalized coordinates rounded to two decimal degrees, together with species identity, locality, date used for chronological ordering, and broad source category. All analyses reported in the manuscript were performed using the original full‐precision coordinates. For historical bibliographic records available only at year‐level temporal resolution, 1 January of the recorded year was used as a sorting placeholder; records sharing the same date retained their original spreadsheet order. This chronology broadly reflects the historical expansion of sampling from a limited set of repeatedly visited areas to wider geographic coverage during the recent citizen‐science period and was used for subsequent analyses of record accumulation.

### 2.3 Inferring Breakpoint‐Derived Grid Cell Size

We used the cells of the following sizes: 1 × 1, 2 × 2, 3 × 3, 4 × 4, 5 × 5, 6 × 6, 7 × 7, 8 × 8, 10 × 10, 12 × 12, 15 × 15, 20 × 20, and 25 × 25 km. We first reprojected the geographic coordinates of the records into the UTM projection (UTM 38N; EPSG:32638); then we set grids of different sizes for Georgia's area and assigned each point to a grid cell. Hence, we counted the total number of grid cells for each studied species and for 13 different cell sizes. The entire workflow in R is shown in Appendix S1.

**FIGURE 1:**
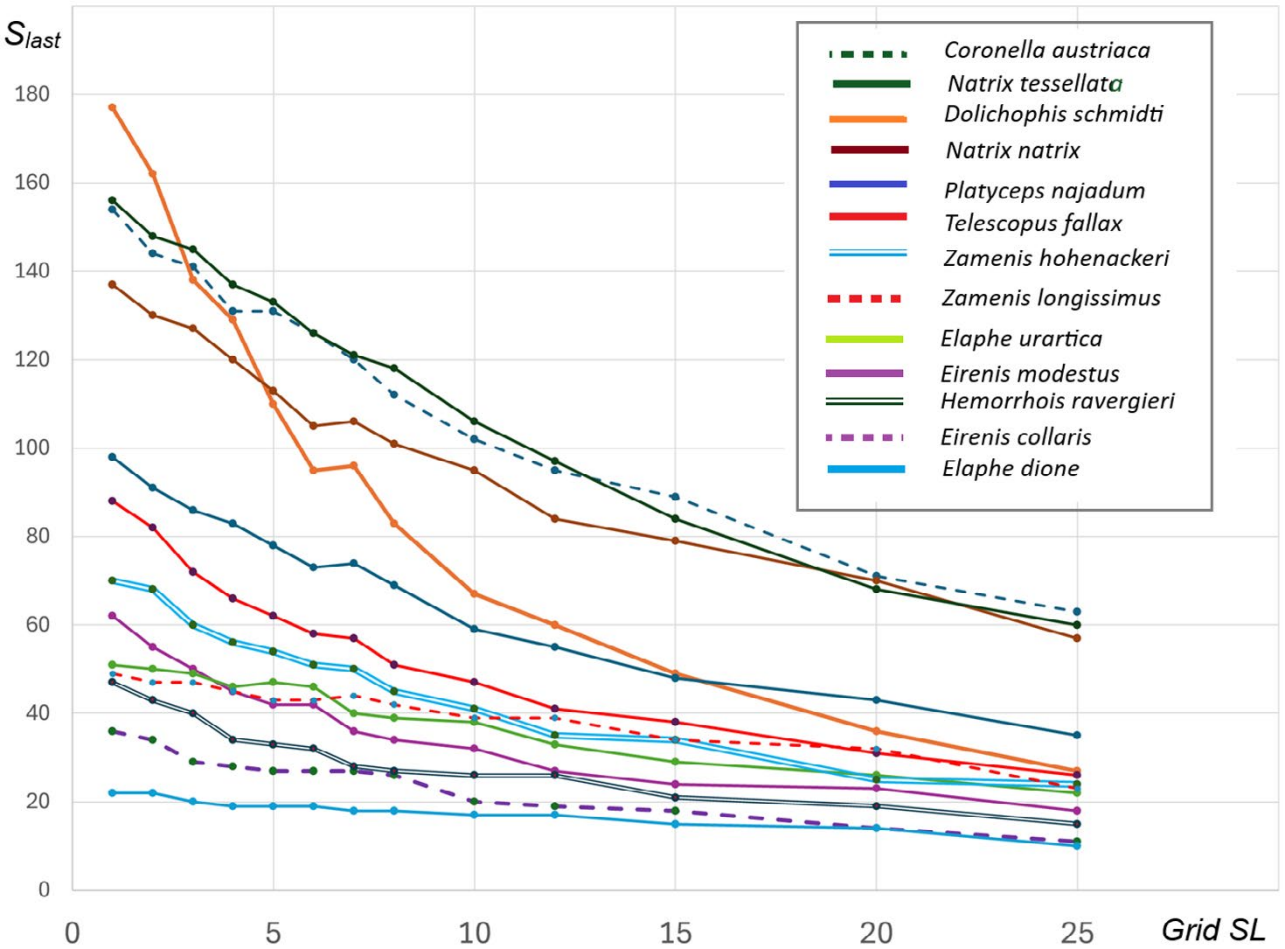
Dependence of the number of grid cells where a species has been recorded in Georgia, *S*_last_ (given the empirical dataset) on the grid SL between 1 and 25 km.

For identifying the breakpoint‐derived grid‐cell size, for each species and based on the total number of records, we analyzed the relationship between grid resolution and the number of occupied cells (*S*_last_; Figure 1). If the set of the known records is representative for describing a species' range, we expect that as cell size increases, the total number of occupied cells will generally decline as increasingly large cells aggregate spatially proximate records. We identified the breakpoint cell size (*ψ*) at which *S*_last_ showed a marked change in slope. We interpret this breakpoint as an informative spatial scale at which occupied‐cell counts become less sensitive to further increases in grid size. The breakpoint itself does not demonstrate that this grain minimizes biological bias or represents the true spatial grain of occupancy. For this purpose, we fitted an initial linear model and then applied the Iterative Least Squares method (V. M. R. Muggeo 2003) to estimate *ψ* and the corresponding slopes for each segment. The analysis was performed in R version 4.5.1 using the *segmented* package v.2.2‐1 (V. M. Muggeo 2008). The significance of the breakpoint was confirmed using Davies (1987) test and Slope Change (Δ slope), with Davies test significance assessed at *p* < 0.05, supplemented by the change in slope (Δ slope) as a descriptive measure of effect size.

### 2.4 Calculating Parameters of the Curves and Saturation Levels

To estimate the asymptotic number of grid cells where a species is likely to occur, we estimated the parameters of the curves describing the increase of the cumulative number of occupied cells *S*(*n*) with the number of the chronologically ordered records. For this purpose, we used three models commonly applied to the analysis of growth slowing with increasing sampling effort (cumulative number of records, hereinafter n) and asymptotically approaching a saturation level *S*_max_. For numerical fitting, record number was standardized within each species × grid‐size accumulation curve as *n*_*sc*_ *= n*/*n*_max_, so that the final empirical observation had *n*_*sc*_ *= 1*. The equations below were fitted using *n*_*sc*_ in place of *n*. This rescaling does not affect the estimated *S*_max_, but the shape parameters *p* and *k* are expressed on the standardized sampling scale. The three equations were:

1. Negative exponential equation (EXP)
(1.1) $$S\left(n\right)=S_{\max}\left(1-e^{-\mathrm{pn}}\right)$$
  where *S*(*n*) is the cumulative number of occupied cells, and *S*_max_ is the saturation level. *p* is a dimensionless scale parameter controlling how rapidly the accumulation curve approaches *S*_max_ across the standardized sampling interval. For the negative exponential model, 1/*p* corresponds to the standardized fraction of the complete empirical sample required to reach approximately 1 − e^−1^ ~ 63% of *S*_max_.
2. Michaelis–Menten equation (rectangular hyperbola; MM)
(1.2) $$S\left(n\right)=S_{\max}\frac{n}{k+n}$$
  where *k* is the standardized record fraction at which *S*(*n*_*sc*_) *= S*_max_/2; in terms of the original record number, this corresponds to *kn*_max_ records.
3. Weibull saturation (generalized exponential; WB)
(1.3) $$S\left(n\right)=S_{\max}\left(1-\exp\;\left(-{\left(\mathrm{pn}\right)}^{c}\right)\right)$$
  Assuming the model becomes exponential when *c* = 1, it approaches the asymptote more slowly when *c* < 1 and more quickly when *c* > 1. The initial parameter values and parameter bounds used for fitting the three saturation models are summarized in Table 1. For each model, fitting was attempted sequentially from the listed starting values until convergence was achieved, while parameter estimates were allowed to vary continuously within the specified bounds.

**TABLE 1:** Initial parameter values and parameter bounds used for fitting the three saturation models.

Model	Parameter	Initial values	Lower bound	Upper bound
Negative exponential (EXP)	*S*_max_	1.3*S*_last_	*S*_last_	80*S*_last_
	*p*	1, 3, 8	10–6	80
Michaelis–Menten (MM)	*S*_max_	1.3*S*_last_	*S*_last_	80*S*_last_
	*k*	0.05, 0.20, 0.50	10–6	20
Weibull saturation (WB)	*S*_max_	1.5*S*_last_	*S*_last_	80*S*_last_
	*p*	2, 4, 8	10–6	80
	*c*	0.7, 1.0, 1.3	0.2	3

*Note:* *S*_last_ is the maximum observed cumulative number of occupied cells for the respective species and grid size. Multiple starting values were used to reduce dependence of nonlinear optimization on initial conditions; parameters were subsequently estimated continuously within the indicated bounds. The bounds for *p* and *k* refer to the standardized record‐number scale *n*_*sc*_ *= n*/*n*_max_.

Because *S*(*n*) is cumulative, successive values of the response are not statistically independent in the usual regression sense. We therefore treated the three equations as descriptive saturation functions. For each species and grid resolution, the models were fitted to the chronologically ordered accumulation curve and the resulting *S*_max_ values were recorded. AIC was used as a pragmatic criterion for ranking the three candidate functions fitted to the same accumulation curve. The model with the lowest AIC was used as the primary model‐derived estimate. The corresponding AOO was calculated as *S*_max_ multiplied by grid‐cell area. Sample completeness was calculated as follows:

(1.4) $$C=\frac{S_{\text{last}}}{S_{\max}}$$

where *S*_max_ refers to the estimate from the lowest‐AIC model. Sensitivity of *S*_max_ to the historical order of occurrence records was evaluated separately by record‐order permutation as described below.

AOO estimates obtained at breakpoint‐derived species‐specific grid sizes were treated as complementary model‐derived estimates. The 2 km grid was analyzed separately to allow direct comparison with the IUCN reference grain.

### 2.5 Calculating Extent of Occurrence (EOO)

For each studied species, we estimated EOO by inferring a minimum convex polygon from the known Georgian records in QGIS.

The entire workflow, including R scripts and the related packages is described in Appendix S2.

### 2.6 Hold‐Out Subsampling Validation

To evaluate the predictive performance of the saturation‐curve approach, we conducted a repeated random subsampling analysis using the six species represented by the largest numbers of occurrence records: *D. schmidti* (201 records), *N. tessellata* (163), *C. austriaca* (162), *N. natrix* (143), *P. najadum* (106), and *T. fallax* (95). For each species, we randomly retained 25%, 50%, or 75% of the complete occurrence dataset without replacement. The selected records were restored to their original chronological order, cumulative occupied‐cell curves were reconstructed for each of the 13 grid sizes, and the EXP, MM, and WB models described above were fitted using the same parameterization as in the primary analysis. One hundred random subsamples were generated for each species and sampling fraction.

For each replicate, we tested whether the model fitted to the reduced dataset could predict the number of occupied cells documented when the number of records reached that of the complete empirical dataset. Because record number was scaled relative to the size of each subsample during model fitting, the fitted model was evaluated at the ratio *n*_full_/*n*_sub_, where *n*_sub_ and *n*_full_ are the numbers of records in the subsample and complete dataset, respectively. The model with the lowest AIC was used for this comparison.

Predictive performance was quantified by comparing the relative error of the raw subsample count,

(1.5) $$E_{\mathrm{raw}}=\frac{|S_{\mathrm{sub}}-S_{\text{full}}|}{S_{\text{full}}}$$

with the relative error of the model prediction,

(1.6) $$E_{\text{pred}}=\frac{|S_{\text{pred}}-S_{\text{full}}|}{S_{\text{full}}}$$

where *S*_sub_ is the observed number of occupied cells in the reduced sample, *S*_full_ is the number documented by the complete empirical dataset, and *S*_pred_ is the value predicted by the fitted accumulation curve at the complete sample size. We also recorded the proportion of replicates in which the model prediction was closer to *S*_full_ than the raw subsample count.

### 2.7 Sensitivity to Record Order

Because the occurrence records accumulated through changing sampling and reporting networks, we evaluated the sensitivity of saturation estimates to record order. For each species, the complete set of records was randomly permuted 500 times without replacement, so that every permutation contained exactly the same records and occupied cells as the empirical dataset but in a different sequence. Within a permutation, the same randomized record order was used across all grid sizes. Cumulative occupied‐cell curves were reconstructed and the EXP, MM, and WB models were refitted.

The *S*_max_ obtained from the actual chronological sequence was retained as the empirical point estimate. To isolate sensitivity to record order from sensitivity to model selection, the model selected by lowest AIC from the chronological dataset was held fixed when calculating the primary permutation distribution. The 2.5th and 97.5th percentiles of this distribution were interpreted as 95% record‐order sensitivity limits. These limits quantify sensitivity of the fitted asymptote to record order and are not interpreted as confidence intervals for AOO. We additionally recorded the proportion of permutations in which the same model had the lowest AIC as in the chronological analysis.

Because each permutation contains every record exactly once, the resulting accumulation curves are non‐decreasing, integer‐valued, satisfy *S*(*n*) *≤ n*, and terminate at exactly the same *S*_last_ as the chronological curve. The breakpoint grid size *ψ* is consequently unaffected by record order because it depends on the final *S*_last_ at each grid resolution. The permutations were used as a sensitivity test rather than as an alternative to the empirical chronology because the chronological sequence reflects the real historical expansion of observer coverage and distributional knowledge. In all three models, *S*_max_ was constrained to be at least as large as *S*_last_, the number of occupied cells already documented in the empirical dataset.

OpenAI ChatGPT was used to assist in developing and debugging the R workflow and in discussing candidate mathematical functions for describing saturation of cumulative occupied‐cell curves. The selection, implementation, evaluation, and interpretation of the statistical models were made by the authors.

## 3 Results

### 3.1 Calculating Breakpoint‐Derived Grid Cell Size

Nine of the thirteen studied species showed significant breakpoints *ψ*, ranging from approximately 4.5–13.0 km (Table 2). The curves linking the grid‐cell size and total number of occupied cells *S*_last_ are shown in Figure 1.

**TABLE 2:** Estimated breakpoint (grid‐cell size *ψ*) of the curves shown in Figure 1.

Species	*ψ*	*ψ* low	*ψ* high	t_value	Davies *p*	Slope before	Slope after	Adj_R2
*Coronella austriaca*	13.016	10.016	16.016	5.258	0.002	−5.414	−2.100	0.984
*Dolichophis schmidti*	8.592	7.683	9.500	13.138	0.000	−13.833	−2.465	0.989
*Eirenis collaris*	11.999	4.969	19.030	2.111	0.168	−1.177	−0.554	0.919
*Elaphe dione*	4.462	3.498	5.427	5.114	0.000	−6.500	−1.105	0.966
*Eirenis modestus*	4.000	−0.702	8.702	0.940	0.307	−1.222	−0.268	0.744
*Elaphe urartica*	18.066	12.197	23.934	1.933	0.114	−1.463	−0.800	0.985
*Hemorrhois ravergieri*	8.734	6.531	10.938	5.450	0.001	−2.381	−0.576	0.955
*Natrix natrix*	10.308	8.796	11.820	9.603	0.000	−5.255	−1.878	0.992
*Natrix tessellata*	10.493	8.476	12.509	7.200	0.000	−5.647	−2.612	0.991
*Platyceps najadum*	10.187	8.426	11.948	8.249	0.000	−4.139	−1.357	0.987
*Telescopus fallax*	6.248	5.159	7.338	7.146	0.000	−6.486	−1.566	0.978
*Zamenis hohenackeri*	11.235	9.454	13.016	8.200	0.000	−3.205	−0.724	0.982
*Zamenis longissimus*	10.000	0.518	19.482	1.360	0.477	−1.133	−0.737	0.937

*Note:* *ψ low* and *ψ high* are 95% confidence limits of the breakpoint estimate. Davies *p* was used to assess breakpoint significance (*p* < 0.05); the *t* value for the slope‐change term is reported as an additional descriptive measure.

Eleven out of the thirteen studied species showed significant breakpoints *ψ* with grid SL varying between 3 and 14 km (Table 2).

### 3.2 Estimating Smax and AOO

Model‐derived estimates of *S*_max_ and AOO for the three candidate saturation functions across species and grid sizes are presented in Table S1. All three models converged successfully for all 169 species × grid‐size combinations. The lowest AIC was obtained by the EXP model in 14.8% of cases, by MM in 23.1%, and by WB in 62.1%, indicating that no single saturation function was universally preferred, although the Weibull formulation was selected most frequently.

At the tested grid sizes nearest to the significant breakpoints, the fitted asymptotes generally exceeded the number of occupied cells documented in the empirical datasets, indicating varying degrees of sampling incompleteness. Median sample completeness, expressed as *C* = *S*_last_/*S*_max_, was 0.60 across the nine species with significant breakpoints. Five of the nine species had *C* ≥ 0.5, whereas one had *C* < 0.25. These results show that the extent to which the empirical occupied‐cell count approached the fitted saturation level differed substantially among datasets.

### 3.3 Validation by Repeated Subsampling

**FIGURE 2:**
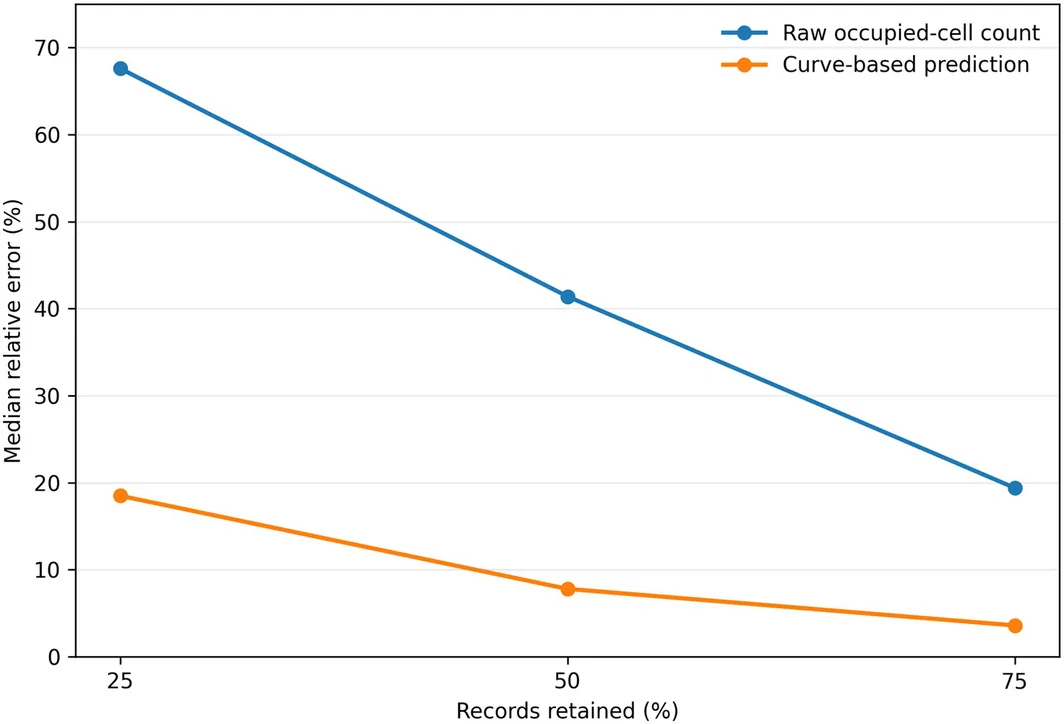
Predictive performance of the saturation‐curve approach in repeated random subsampling of the six best‐represented species. Lines show the median relative error of the raw occupied‐cell count and the model‐based prediction when 25%, 50%, or 75% of occurrence records were retained. Curve‐based predictions were closer to the complete‐data occupied‐cell counts than raw incomplete counts in 97.3%, 97.8%, and 96.3% of comparisons, respectively.

Repeated subsampling strongly supported the predictive performance of the saturation‐curve approach (Table 3; Figure 2). When only 25% of the occurrence records were retained, the median relative error of the raw occupied‐cell count was 0.676, whereas the median error of the curve‐based prediction was 0.185. When 50% of records were retained, the corresponding errors were 0.414 and 0.078, and when 75% were retained, they were 0.194 and 0.036, respectively. Curve‐based predictions were closer to the occupied‐cell counts documented by the complete empirical datasets than the raw incomplete counts in 97.3%, 97.8%, and 96.3% of comparisons at the three sampling fractions, respectively. Median prediction bias was slightly negative (−0.047, −0.021, and −0.014, respectively), indicating a small tendency toward underprediction rather than systematic overprediction. All 70,200 nonlinear model fits performed during the validation converged successfully.

**TABLE 3:** Performance of saturation‐curve predictions in repeated random subsampling of the six best‐represented species.

Records retained (%)	Median raw error (%)	Median prediction error (%)	Median prediction bias (%)	Prediction better than raw count (%)
25	67.6	18.5	−4.7	97.3
50	41.4	7.8	−2.1	97.8
75	19.4	3.6	−1.4	96.3

The tabulated results of the curve analysis for 13 studied species are shown in Table S1. At the 2 km grid size, *S*_last_ varied from 20 occupied cells in *E. dione* to 165 in *D. schmidti* .

The observed and model‐derived AOO estimates at the 2 × 2 km reference grain are presented in Table 4. Observed record‐based AOO ranged from 80 to 660 km^2^, whereas the corresponding model‐derived AOO estimates ranged from 143 to 6941 km^2^. In all species, the model‐derived estimate exceeded the AOO calculated directly from the documented occupied cells.

**TABLE 4:** Observed and model‐derived area of occupancy (AOO) for the 13 studied species using a 2 × 2 km grid.

Species	*S*_last_	Observed AOO (km^2^)	Model	*S*_max_	Model‐derived AOO (km^2^)	*C*
*Dolichophis schmidti*	165	660	WB	758	3034	0.22
*Natrix tessellata*	151	604	EXP	1009	4034	0.15
*Coronella austriaca*	148	592	MM	1377	5510	0.11
*Natrix natrix*	129	516	WB	376	1502	0.34
*Platyceps najadum*	91	364	EXP	1735	6941	0.05
*Telescopus fallax*	82	328	WB	657	2630	0.12
*Zamenis hohenackeri*	66	264	WB	1344	5377	0.05
*Eirenis modestus*	55	220	WB	828	3312	0.07
*Elaphe urartica*	49	196	EXP	897	3589	0.05
*Zamenis longissimus*	47	188	EXP	1356	5422	0.03
*Hemorrhois ravergieri*	42	168	WB	68	270	0.62
*Eirenis collaris*	31	124	WB	644	2575	0.05
*Elaphe dione*	20	80	WB	36	143	0.56

*Note:* *S*_last_ is the number of occupied cells documented in the complete empirical dataset; observed AOO is 4 *S*_last_. *S*_max_ and model‐derived AOO are estimates from the saturation model with the lowest AIC for the chronological accumulation curve. *C* = *S*_last_/*S*_max_. In all models, *S*_max_ was constrained to *S*_max_ ≥ *S*_last_.

Abbreviations: EXP, negative exponential; MM, Michaelis–Menten; WB, Weibull saturation.

### 3.4 Sensitivity to Record Order

All 253,500 nonlinear fits to the 500 permuted record sequences converged successfully. Because accumulation curves were reconstructed directly from permuted occurrence records, all were non‐decreasing, integer‐valued, satisfied *S*(*n*) ≤ *n*, and terminated at the same *S*_last_ as the corresponding chronological curve. At the breakpoint‐derived grid sizes, the median absolute difference between the chronological *S*_max_ and the median obtained after record‐order permutation was 28.0%. The chronological *S*_max_ fell within the 2.5th–97.5th percentile permutation range for 8 of the 9 species with significant breakpoints. The same lowest‐AIC model as in the chronological dataset was recovered in a median of 62.2% of permutations.

## 4 Discussion

Our results indicate that the standard 2 km grid may yield strongly conservative AOO estimates. This applies to many species represented by sparse, spatially uneven, or recently expanding record sets. In the snake dataset analyzed here, nine of the 13 species showed significant breakpoints at coarser resolutions, ranging from approximately 4–13 km. This finding suggests that larger grid sizes commonly provide more stable accumulation behavior. We view the proposed workflow as a complementary tool for evaluating scale dependence and the sensitivity of AOO estimates to incomplete sampling. Accordingly, breakpoint‐derived AOO estimates provide additional information on how incomplete sampling and spatial resolution influence occurrence‐based AOO estimates.

The repeated subsampling analysis provided direct support for the predictive performance of the accumulation‐curve approach: model‐based predictions consistently outperformed raw occupied‐cell counts and became increasingly accurate as sampling completeness increased.

Chronological accumulation also reflects changes in the spatial structure of sampling. Early professional records were commonly concentrated around major cities and intensively studied areas; subsequent professional surveys expanded coverage to additional areas, whereas recent records from amateur naturalists and online platforms substantially broadened geographic coverage and documented isolated localities in regions where some species were not recorded earlier. The permutation analysis confirmed that this historical sequence can influence fitted *S*_max_, although the magnitude and direction of the effect differed among species. We therefore retain the chronological sequence as the empirical history of increasing distributional knowledge and use random permutations only to quantify sensitivity to record order. Importantly, sensitivity to record order did not diminish the predictive performance demonstrated by the hold‐out analysis, in which accumulation‐curve predictions outperformed observed incomplete occupied‐cell counts in 96%–98% of comparisons. Selection of the appropriate grid‐cell size is an important issue not only for estimating the AOO of individual species, but also for choosing appropriate data resolution in spatial modeling. Guisan and Thuiller (2005), in their discussion of constraints in spatial modeling, expressed concern about the use of overly large grid cells for developing spatial models. Seo et al. (2009) likewise discouraged the use of excessively large grid cells (e.g., 50 × 50 km, commonly used in global‐scale plant studies), because these may lead to substantial overestimation of species' true ranges inferred from spatial modeling. They also showed that narrow‐ranged plant species are more sensitive to grid size than widespread ones. In both cases, the authors emphasized the advantages of using relatively small grid cells and the most precise possible occurrence data. Budic et al. (2016) further suggested that increasing cell size in spatial modeling of ungulate distributions leads to greater discrepancies between cells identified from geographic and projected coordinates. These studies mainly highlight the potential bias associated with overly coarse grids. On the other hand, Maciel and Guilherme (2022) suggested for tree species that coarser resolutions may be more suitable for predicting species–abundance–density relationships. Marsh et al. (2018, 2019) used computer simulation models to suggest that downscaling can be a useful tool for estimating AOO in IUCN Red List assessments, especially when sampling coverage is low. More generally, the search for a balance between coarse and fine geographic resolution has been the subject of intensive discussion during the last decade (Azaele et al. 2012; Chao and Jost 2012; Barwell et al. 2014).

Whereas researchers who use spatial data for distribution modeling discuss the issue of optimal grid size, the IUCN, to maintain consistency, explicitly requires the use of a 2 km grid (4 km^2^ per cell) for estimating AOO (IUCN Standards and Petitions Committee 2024). The IUCN states that this specific size was chosen as fine enough to capture the habitat of narrow‐range endemics, and coarse enough to account for some level of positional uncertainty in occurrence data. It is suggested that, since AOO increases with grid size (the “scale‐area relationship”: see Hui 2009), using a standardized cell size prevents species from being classified into different threat categories if researchers choose a different map resolution. Even if a species has a very small or a huge home range, the assessment must still use the 2 km grid for the formal Red List criteria. These strict approaches, on the one hand, appear to standardize vulnerability estimates across researchers and species; however, IUCN acknowledges that, in cases of too sparse data, the estimated AOO should be taken with care due to potential bias. We suggest that they may lead to *unacceptably strong* bias. In fact, a reliable estimate of AOO is possible only if the number of grid cells with species presence asymptotically approaches *S*_max_, beyond which discoveries will not increase the number of occupied cells. This may be evident from the presence of a breakpoint in a curve, connecting the number of occupied cells with the number of findings; obviously, such a breakpoint is easier to achieve on a coarse geographic scale. On the other hand, too coarse a scale may lead to overestimation of AOO, because a substantial part of a large occupied cell may remain unsuitable for the species. The breakpoint used here therefore represents a practical scale at which the sensitivity of occupied‐cell counts to increasing cell size is reduced. We suggest that a compromise approach can yield the most reliable results and is straightforward to implement and evaluate with a simple statistical procedure.

For species used in this study, the average EOO/AOO ratio was estimated to be 7.6, suggesting the absence of species from a large part of their extent of occurrence. This ratio was especially high in species with strict requirements for environmental conditions, such as dwarf snakes (*Eirenis*) and the spotted whipsnake ( *H. ravergieri* ). The Weibull model had the lowest AIC most frequently in the chronological analyses. However, record‐order permutations showed that model selection was moderately sensitive to the sequence of observations, and we therefore interpret differences among the candidate saturation functions descriptively rather than as evidence for a single general form of occurrence accumulation. The chronological accumulation patterns also reflect the historical structure of sampling: early findings of most Colubrid species were concentrated in limited areas, including the surroundings of large cities and the areas favored by professional herpetologists; hence, early aggregation of new findings did not substantially increase the area where the presence of species was established. More recent findings, rapidly increasing with the expansion of social media and specialized web resources, as well as the number of amateur naturalists in Georgia, have led to the faster expansion of documented occupancy as geographic coverage broadened.

Anderson (2023) suggested that the fixed 2 km criterion was selected to balance the resolution needed for narrow‐ranged species with the wider data commonly available for species with broad ranges. He proposed complementing occurrence‐based estimates with spatial modeling and prevalence information, thereby combining empirical occurrence records, including those derived from citizen science, with predictions of suitable habitat at the 2 × 2 km grid‐cell scale. Araújo et al. (2019) called for a broader consensus on developing standard spatial modeling approaches that could substantially improve the comparability of data from researchers worldwide. We agree that properly applied spatial modeling will help to avoid substantial underestimation of AOO. However, since the IUCN reasoning involves standardizing results across very different species and researchers with individual, uncoordinated approaches, this may complicate interpretation: different researchers use different spatial modeling methods and different predictors of habitat suitability, and this will be difficult to overcome due to both subjective preferences and to taxon‐specific modeling, relying on different predictors and modeling algorithms.

The idea of assuming completeness of the species' records while inferring size of a species range is not new (see e.g., Chao and Jost 2012). The approach we use in this paper helps to avoid this uncertainty and subjectivity in the estimation of the occupied ranges, being dependent solely on the empirically established records and avoiding any unnecessary assumptions, except the assumption that the increase of the cumulative number of occupied cells is limited by the real size of the area where a species occurs. Adjusting grid‐cell size may be an unnecessary specification for estimating AOO of trees, large birds, or ungulate mammals, hence those species whose ranges we can assume to be sufficiently well‐known. However, for smaller animals, we often lack sufficient information to reconstruct their ranges precisely, and each new finding may substantially increase both AOO and EOO for a species. The evaluation of the status of Syrian spadefoot toads in Georgia provides a good example of how existing findings can underestimate a species' range. Before field research conducted over several years by I. Serbinova, the known number of sites, collected by scientists since the beginning of the 20th century, was below 10, and the species was considered vulnerable in Georgia. However, a precise analysis of the range, based on the identification of tadpoles at breeding sites, increased the number of sites to 13 and led to a substantial increase in the known EOO and AOO of this species (Tarkhnishvili et al. 2009).

The workflow proposed here, although it requires additional computational effort beyond recording a species' presence in a grid cell, is easy to implement and replicable and does not require specialized knowledge or subjective ideas about limiting factors or preferred spatial modeling approaches. It helps evaluate model‐derived AOO while providing a way to quantify both sampling incompleteness and sensitivity of the resulting estimates to the historical sequence of occurrence records. Finally, spatial modeling can further improve the knowledge: if modeling applies to the grid cells where species' presence is established or highly expected (even in case of large grid cells, such as 15 × 15 km), the outcome of the modeling even with a simple algorithm, for example, calculating the area within the presence cells covered with a landscape appropriate for a species (e.g., based on NDVI classification), can yield precise estimation of the range.

## Author Contributions

**Giorgi Iankoshvili:** conceptualization (equal), data curation (lead), formal analysis (supporting), investigation (lead), methodology (supporting), project administration (lead), resources (lead), writing – original draft (supporting). **David Tarkhnishvili:** conceptualization (equal), formal analysis (lead), methodology (lead), project administration (supporting), writing – original draft (lead).

## Funding

This work was supported by Shota Rustaveli National Science Foundation (grant # FR‐23‐17324).

## Conflicts of Interest

The authors declare no conflicts of interest.

## Supporting information


**Table S1:** Model‐based estimates of saturation level and area of occupancy (AOO) for 13 snake species across 13 grid‐cell sizes. For each species × grid‐size combination, *S*_last_ is the observed number of occupied cells. EXP, MM and WB columns report the fitted *S*_max_ and corresponding AOO estimates for the three candidate saturation models. The table additionally identifies the lowest‐AIC model, its *S*_max_ and AOO estimate, and sample completeness (*C* = *S*_last_/*S*_max_).

**Table S2:** Hold‐out subsampling validation of saturation‐curve predictions for six well‐represented snake species across 13 grid‐cell sizes. For each species, 25%, 50%, or 75% of occurrence records were randomly retained without replacement in 100 replicate subsamples. S_full is the number of occupied cells documented from the complete empirical dataset, median_S_sub is the median number documented in the reduced datasets, and median_S_pred_full is the median occupied‐cell number predicted at the complete empirical sample size by the lowest‐AIC saturation model. pred_low95 and pred_high95 are the 2.5th and 97.5th percentiles of predictions among random subsamples. median_raw_rel_error and median_prediction_rel_error are the median relative errors of the raw subsample count and model‐based prediction, respectively; median_prediction_bias is the signed relative prediction error; median_improvement is the reduction in relative error obtained by model prediction; and prop_prediction_better is the proportion of replicates in which the model prediction was closer to the complete‐data occupied‐cell count than the raw subsample count.

**Table S3:** Record‐order sensitivity analysis at the breakpoint‐derived grid sizes for the nine species with significant breakpoints. The table reports the breakpoint estimate, nearest tested grid size, chronologically selected model, chronological *S*_max_, permutation median and 2.5th–97.5th percentile sensitivity limits, and the proportion of permutations recovering the same lowest‐AIC model.

**Appendix S1:** R script for (i) reprojecting occurrence coordinates to UTM, (ii) building grids at 13 cell sizes, and (iii) computing cumulative numbers of occupied grid cells through time for each species.

**Appendix S2:** R script for fitting the three saturation models (negative exponential, Michaelis–Menten and Weibull), estimating *S*_max_ and model‐derived AOO, selecting the lowest‐AIC model, calculating sample completeness, performing the record‐order sensitivity analysis, and estimating breakpoint‐derived grid sizes.

**Appendix S3:** R script implementing the repeated random subsampling validation of the saturation‐curve approach. The script randomly retains 25%, 50%, or 75% of occurrence records for the six best‐represented species, restores the retained records to their original chronological order, reconstructs cumulative occupied‐cell curves at 13 grid sizes, fits negative exponential, Michaelis–Menten, and Weibull saturation models, selects the lowest‐AIC model, and predicts the number of occupied cells at the complete empirical sample size. It also calculates prediction error, bias, improvement relative to raw incomplete occupied‐cell counts, model‐fitting diagnostics, and the summary statistics reported in Table 3 and Table S2.

**Data S1:** Occurrence dataset for the 13 snake species analyzed in this study, including species identity, generalized locality coordinates, date used for chronological ordering, broad source category, and bibliographic source or observer name. For conservation reasons, publicly released coordinates are rounded to two decimal degrees (approximately 1‐km spatial resolution); analyses were performed using the original full‐precision coordinates.

## Data Availability

The occurrence dataset underlying the analyses is provided with this article as Supporting Information S1. To reduce risks associated with public disclosure of precise animal localities, geographic coordinates in the public dataset have been rounded to two decimal degrees; all analyses were conducted using the original full‐precision coordinates. Full‐precision coordinates can be made available to the journal editors or reviewers for verification and to qualified researchers upon justified request. Detailed analytical results are provided in Tables S1–, S3, and the R scripts implementing the analyses are provided in Appendices S1–, S3.
